# Compassionate Use of Osocimab in Preventing Thrombotic Complications Without Incremental Bleeding: A Case Report

**DOI:** 10.1055/a-2577-4474

**Published:** 2025-05-06

**Authors:** Jan Beyer-Westendorf, Katrin Weber, Falk Eckart, Martin W. Laass, Ralf Knöfler, Kate Benson, László B. Tankó, Martin Bornhäuser

**Affiliations:** 1 Department of Medicine 1, University Hospital Carl Gustav Carus, Technische Universität Dresden, Dresden, Germany; 2 Department of Pediatrics, University Hospital Carl Gustav Carus, Technische Universität Dresden, Dresden, Germany; 3 Bayer Pharmaceuticals, Reading, United Kingdom; 4 Bayer Pharmaceuticals, Basel, Switzerland

**Keywords:** anticoagulation, catheter-related thrombosis, factor Xia, gastrointestinal bleeding, parenteral feeding, osocimab

## Abstract

**Objective:**

To describe an innovative anticoagulation strategy in a 20-year-old woman with innate jejunal atresia and ultrashort bowel syndrome who was dependent on long-term parenteral nutrition and suffered from multiple venous thrombotic events and bleeding complications since infancy.

**Design:**

Single-patient case report.

**Setting:**

Dresden University Hospital, Dresden, Germany.

**Patient:**

Being fully CVC-dependent since birth, our patient repeatedly developed catheter-related thrombosis (CRT) since infancy and was treated with daily low-molecular-weight heparin injections for more than 15 years. Despite this, clotting, severe gastrointestinal bleeding, and osteoporosis remained a persistent problem, causing numerous hospitalizations over the years, significant developmental delays, and a decline in the patient's body mass index (BMI). A short period of rivaroxaban treatment had to be stopped owing to acute gastrointestinal bleeding. After the failure of all approved anticoagulant concepts, compassionate use access was granted to the investigational drug osocimab, a human monoclonal antibody inhibitor of factor XIa. Hereditary FXI deficiency as well as FXI inhibition in animal models have been shown to reduce arterial and venous thrombosis without increasing bleeding. Consistent with this, short-term osocimab treatment has shown clinical efficacy in preventing postoperative venous thromboembolism after knee replacement surgery and in reducing dialysis conduit clotting compared with placebo in patients undergoing hemodialysis, without increasing the rate of clinically relevant bleeding versus comparators. After initiating osocimab, the patient experienced no further clotting complications, and bleeding decreased in frequency and severity. The patient's BMI decline immediately stopped; her weight increased by over 10% in the subsequent 20 months, and menstruation started 3 months later without signs of menorrhagia. Now, with 2.5 years of uninterrupted exposure outside of a clinical trial, this patient has experienced the longest duration of factor XIa inhibition to date. She continues to receive osocimab under the compassionate use program and maintains a positive change in her well-being and quality of life.

## Introduction

Central venous catheter (CVC) thrombosis is typically managed by CVC removal and short-term anticoagulation therapy; however, for patients with lifelong CVC dependency management is difficult, particularly for those who also have a high bleeding risk. Here, we report the successful management of a 20-year-old woman with ultra-short bowel syndrome (uSBS) who was dependent on CVCs for parenteral nutrition (PN) and experienced multiple catheter-related thromboses and bleeding complications throughout her life.

Our patient has jejuno-ileal atresia, a congenital malformation of the small intestine, which has an estimated prevalence of 0.7/10,000 live births in Europe. 1 Apple-peel syndrome (type IIIb intestinal atresia), characterized by innate jejunal atresia and the small intestine wrapping spirally around supplying blood vessels, belongs to the most severe forms and comprises less than 10% of jejuno-ileal atresia cases. 2 3 4 Patients with apple peel syndrome often have complex presentations with various comorbidities. 4 5

Despite the patient receiving daily low-molecular-weight heparin (LMWH; enoxaparin) injections for more than 15 years since infancy to prevent or treat catheter-related thrombosis (CRT), clotting of CVCs, CRT, and severe gastrointestinal bleeding remained a persistent problem, along with repeated catheter replacements and severe osteoporosis.

## Patient Clinical History

**Fig. 1:**
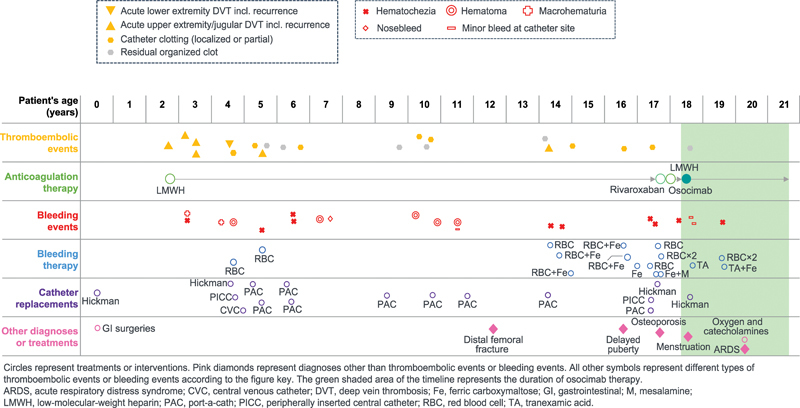
Timeline of patient's medical history, including thrombotic events, bleeding events, and treatments. Circles represent treatments or interventions. Pink diamonds represent diagnoses other than thromboembolic events or bleeding events. All other symbols represent different types of thromboembolic events or bleeding events according to the figure key. The green shaded area of the timeline represents the duration of osocimab therapy. ARDS, acute respiratory distress syndrome; CVC, central venous catheter; DVT, deep vein thrombosis; Fe, ferric carboxymaltose; GI, gastrointestinal; LMWH, low-molecular-weight heparin; M, mesalamine; PAC, port-a-cath; PICC, peripherally inserted central catheter; RBC, red blood cells; TA, tranexamic acid.

After diagnosis of apple–peel syndrome at birth, sections from the patient's intestines were removed surgically by repeated resections and anastomoses, leading to uSBS and near-complete loss of intestinal function. Subsequent Z-incision surgeries failed to extend the gut and scarred the small intestine, which later continued to be a recurrent source of gastrointestinal bleeding events. 6 To provide PN, a Hickman CVC was inserted into the right jugular vein. Subsequently, the patient experienced recurrent complications of bleeding and thromboembolic events ( Fig. 1 ).

Aged 2 years, the patient developed jugular deep vein thrombosis (DVT) from catheter use and initiated once-daily subcutaneous enoxaparin 10 mg. Jugular DVT recurred twice over the next 12 months, and the patient also experienced subclavian/brachiocephalic DVT. Repeated enoxaparin dose adjustments were undertaken to balance the risk of thrombosis versus bleeding. Aged 4 years, she experienced gastrointestinal bleeding, macrohematuria, and multiple thigh hematomas from enoxaparin injections. Right iliac DVT was discovered—probably caused by a previous temporary CVC—resulting in the first of many CVC replacements. Aged 5 and 6 years, she experienced further CRTs, each requiring CVC replacement; twice-daily enoxaparin was required to maintain vascular access, which increased bleeding event frequency ( Fig. 1 ). Despite a negative family history of venous thromboembolism, a complete thrombophilia screening was performed at the age of 6 years, which ruled out hereditary or acquired thrombophilia.

Aged 10 years, another CRT led to a further enoxaparin dosage increase (30 mg twice daily). The patient experienced a spontaneous distal femoral fracture at 12 years of age, indicating the advanced stage of osteoporosis, a reported complication of short-bowel syndrome, and a side effect of proton pump inhibitors and LMWHs. 7 8 9 Aged 14 years, magnetic resonance imaging revealed known right jugular and brachiocephalic vein occlusions and a new right subclavian vein occlusion. Persistent iron deficiency anemia from age 14 (>2 years) was treated with intravenous ferric carboxymaltose.

Aged 17 years, a bone density scan quantitatively documented the presence of severe osteoporosis (Z-scores: −2.0 [right femur]; −2.3 [left femur]; −4.2 [lumbar spine]). Height and weight developmental milestones were not achieved and delayed puberty was diagnosed ( Fig. 1 ). Catheter site abscesses and CRT led to a Hickman CVC being inserted into the left jugular vein. The patient refused further enoxaparin injections and trialed rivaroxaban 15 mg, later decreasing the dose to 10 mg; however, persistent gastrointestinal bleeding led to treatment discontinuation after 4 weeks. She briefly resumed enoxaparin but quickly refused further injections.

Following several discussions with the patient and her legal guardian, the clinical team suggested an individual treatment approach with the experimental drug osocimab, a human monoclonal antibody inhibitor of factor XIa. 10

Hereditary FXI deficiency as well as FXI inhibition in animal models have been shown to reduce arterial and venous thrombosis without increasing bleeding. 11 12 13 Based on this rationale, several FXI inhibitors are in clinical development. 14 Within this group, osocimab has shown clinical efficacy versus enoxaparin in preventing postoperative venous thromboembolism in individuals undergoing total knee arthroplasty and in lowering the risk versus placebo of dialysis conduit clotting in patients with kidney failure undergoing regular hemodialysis, without increasing the rate of clinically relevant bleeding. 10 15 16

## Methods

Following approval from the Clinical Ethics Committee of Dresden University Hospital, an application was made to the manufacturer (Bayer AG) for compassionate-use access to osocimab. In November 2021, the patient discontinued enoxaparin and initiated weight-based (1.2 mg/kg) intravenous therapy with osocimab (total dose 55 mg). Out of ethical considerations (compassionate use in an underaged patient), and given the limited evidence available at the time to inform dosage, it was decided to initiate osocimab at 1.2 mg/kg and only escalate if clinically needed.

Owing to the long half-life, osocimab can be administered at monthly intervals. The first three doses were administered during day-long hospital stays for close surveillance; the subsequent 21 doses were administered during regular monthly gastroenterology outpatient visits and with 3-hour observation windows following each infusion.

This case is reported with the Ethics Committee approval (Dresden University Hospital) and in accordance with the Declaration of Helsinki 17 and the CARE Checklist. 18 The patient and her legal guardian provided written informed consent.

## Results

During 30 months of osocimab treatment, no new thrombotic events occurred. The CVC dislocated 2 months after treatment initiation leading to insertion of the current Hickman CVC. During the procedure, sequelae of an old clot (considered unrelated to osocimab) were discovered in the patient's left jugular vein. In the subsequent 28 months, the Hickman CVC has been uncompromised.

Minor bleeding at the catheter site from a minor skin infection occurred after the fourth osocimab infusion; the patient was seen at the hospital and discharged with no further action. A clinically relevant gastrointestinal bleed (considered unrelated to osocimab treatment) occurred between the seventeenth and eighteenth infusions, manifesting as hematochezia for 3 days and anemia (treated with tranexamic acid [600 mg] and 2 units of red blood cells plus ferric carboxymaltose [2 × 500 mg], respectively). After osocimab injections, the patient demonstrated normal to moderately prolonged activated partial thromboplastin times (range: 32–42 seconds; reference range: 24–36 seconds). Factor XI values remained in the normal range during the entire period (range: 78–102%; reference range: 60–150%). There were no signs of intolerance or allergic reactions to osocimab.

Aged 20 years, the patient developed acute respiratory distress syndrome, requiring hospitalization and treatment with high-flow oxygen and low-dose catecholamines. Recurrent venous thromboembolism and pneumonitis were ruled out, and atypical pneumonia was diagnosed, considered unrelated to osocimab. The patient fully recovered and osocimab therapy remained uninterrupted without recurrent respiratory symptoms.

During the 18 months before receiving osocimab, the patient's BMI decreased—probably owing to CRT, recurrent hospitalizations, and psychological burden—leading to a rapid deviation from developmental percentiles ( Table 1 ). After initiating osocimab, the patient's BMI decline immediately stopped; her weight increased by more than 10% in 20 months. Also, menarche occurred 3 months later without signs of menorrhagia. After 24 osocimab infusions, dual-energy X-ray absorptiometry indicated improved lumbar spine (−2.8) and right femur (−1.3) Z-scores but a somewhat worsened left femoral Z-score (−3.0) versus the initial scan. The patient reported a positive change in well-being and quality of life after discontinuing enoxaparin and continues to receive osocimab under the compassionate use program (extended until August 2025). To our knowledge, no similar cases have been reported previously.

**Table 1:** Summary of patient's weight, height, and body mass index from 16 to 20 years of age

Age, y	Weight, kg	Height, cm	Body mass index, kg/m ^2^	Developmental percentile	Treatment notes
16	41.8	146.5	19.5	30th	Enoxaparin
17	42.3	147	19.6	29th	Enoxaparin
17	40.8	148	18.6	15th	Enoxaparin
17	42	149.5	18.8	16th	Return to enoxaparin after 4-wks trial of rivaroxaban
18	42.2	150.3	18.7	14th	Before osocimab dosing
18	45.0	151	19.7	NA	2 mo of osocimab
18	44.7	153	19.1	NA	6 mo of osocimab
19	46.7	153	19.9	NA	10 mo of osocimab
19	47.2	154	19.9	NA	12 mo of osocimab
19	46.2	153	19.7	NA	19 mo of osocimab
19	46.5	154	19.6	NA	20 mo of osocimab
19	45.3	154	19.1	NA	22 mo of osocimab
20	47.5	154	20	NA	30 mo of osocimab

Abbreviation: NA, not applicable.

Note: There are no developmental percentiles in Germany for adults.

## Discussion

This case highlights the therapeutic potential of factor XIa inhibition in preventing CRT. CVCs are used widely in clinical practice to administer fluids, medications (including chemotherapy), and PN, and to perform hemodialysis, but they are associated with both short-term (e.g., infections, sepsis, embolism, thrombus extension) and long-term complications (e.g., postthrombotic syndrome, thrombosis recurrence, stenosis or occlusion, vascular access loss). 19 20 Patients with short bowel syndrome and uSBS must adhere to potentially life-long daily PN via CVCs, so the goal in children is to preserve access sites by minimizing catheter replacement. 21 No anticoagulant drugs are approved for use in children or adults with CRT. 20 22 Children undergoing long-term PN who develop CRT may benefit from prophylactic dosing of LMWH to prevent recurrence 21 ; however, ensuring adherence, balancing the increased bleeding risk, and managing the burden on families can be challenging in children. 21

The rationale for inhibiting factor XI derives from the observation that on contact of blood with surfaces of medical devices, FXII is cleaved into activated FXII, which in turn activates FXI, leading to the formation and propagation of a blood clot. 23 Consequently, inhibition of this contact activation pathway may prevent this frequent complication in CVC patients.

The patient presented here has received osocimab therapy in clinical practice for more than 2 years, with maximum adherence. Her outcomes are consistent with those observed in the recently published CONVERT phase 2b trial evaluating osocimab in patients with kidney failure on regular hemodialysis, the majority of whom regularly received concomitant heparin. 16 The limitations associated with a single case report should be addressed with a randomized controlled trial in patients with CVCs to evaluate quality of life and treatment burden. Of note, a phase 2 trial of the anti-FXI monoclonal antibody gruticibart in 22 individuals with cancer undergoing central line placement found that gruticibart treatment resulted in a lower incidence of catheter-associated thrombosis. 24 In addition, a phase 2 study investigating the efficacy and safety of two FXI antibodies, REGN7508 and REGN9933, for preventing blood clots in adults with a peripherally inserted central catheter is currently ongoing (ClinicalTrials.gov: NCT06299111), the results of which may provide further evidence of the benefits of FXI inhibition in these situations.

## Conclusion

These findings suggest that osocimab therapy may be beneficial as part of a multidisciplinary approach to managing conditions that involve CRT. They also highlight the therapeutic potential of factor XIa inhibition in preventing the problems associated with clotting in these patients. With 2.5 years of uninterrupted exposure, this patient has experienced the longest duration of factor XIa inhibition to date, outside of a clinical trial. She continues to report a positive change in well-being and quality of life.
